# Editorial: Oxidative stress and neuroinflammatory responses associated with metal toxicity in brain disorders

**DOI:** 10.3389/fneur.2023.1283653

**Published:** 2023-09-26

**Authors:** M. D. Pandareesh, Mythri Rajeswara Babu, K. Vijayalakshmi, David J. Titus

**Affiliations:** ^1^Department of Biochemistry, Adichunchanagiri School of Natural Sciences, Center for Research and Innovation, Adichunchanagiri University, Mandya, India; ^2^Adichunchanagiri Institute of Medical Sciences, Adichunchanagiri University, Mandya, India; ^3^Department of Psychology, Christ (Deemed to be University), Bangalore, India; ^4^Department of Neurophysiology, National Institute of Mental Health and Neuro Sciences (NIMHANS), Bangalore, India; ^5^Department of Psychiatry and Behavioral Sciences, University of Minnesota, Minnesota, MN, United States

**Keywords:** neuroinflammation, oxidative stress, metal toxicity, neurodegeneration, molecular mechanisms

The intricate interplay between oxidative stress, neuroinflammation, and metal toxicity in several brain disorders has emerged as a compelling avenue of research. This Research Topic aims to understand the contribution of metal toxicity, oxidative stress, neuroinflammation, and their interrelated pathomechanisms to various brain disorders. Each study investigates crucial aspects of their respective domains, shedding light on previously unexplored or inadequately understood areas.

Alzheimer's disease (AD) is marked by an array of distinctive features, including the accumulation of amyloid-beta plaques, neurofibrillary tangles, oxidative stress, neuroinflammation, synaptic impairment, and neurodegeneration. The cascade of events leading to cognitive decline and the mechanisms underlying these processes are yet to be fully elucidated. Metal dysregulation, particularly the excessive release of zinc by presynaptic neurons, has emerged as a critical player in tau pathology and oxidase activation observed in AD.

The complex interplay between molecular mechanisms and neuropathological manifestations in AD continues to captivate neuroscientists. This research topic includes a recent study by Lai et al. that delves into the multifaceted connections between metal dysregulation, the mammalian target of the rapamycin (mTOR) pathway, and AD pathology. The study also explores the role of excessive zinc released by neurons, the activities of mTOR/p70S6K, and the impact of oxidative stress on tau hyperphosphorylation, synaptic impairment, and cognitive decline. With AD's rising prevalence and the ever-increasing need for effective therapeutic strategies, the study holds great promise in advancing our understanding of this devastating disorder.

A pivotal discovery of this study is the elevation of mTOR and ribosomal S6 protein kinase (p70S6K) activities in the brains of patients with AD. Although it is known that the mTOR pathway regulates protein synthesis, degradation, and age-dependent cognitive decline, the precise downstream effectors influenced by mTOR in these contexts remain an intriguing puzzle. The study uncovers the mechanism of mTOR/P70S6K activation mediated mitigation of zinc-induced tau hyperphosphorylation, oxidative stress, tau degeneration, and synaptic and cognitive impairment *in-vivo*. The study demonstrated that rapamycin inactivated the Nrf2/HO-1 pathway and rescued mTOR/p70S6K activation, which could thereby serve as a potential therapeutic target.

Fu et al. addressed the relatively under-recognized impact of air pollution on neurological disorders. While research has long illuminated its devastating consequences on lung and cardiovascular health, the intricate relationship between air pollution and neurological disorders remains less explored and recognized. This article brings to the forefront this underappreciated facet of the harmful effects of air pollution, presenting a comprehensive overview of its impact on the nervous system and neurological diseases.

The Global Burden of Disease report paints a grim picture, attributing a staggering 6.5 million deaths to air pollution in 2015 alone. Air pollution not only causes a spectrum of acute and chronic diseases but also seeps into the realm of neurological health. Central to this discussion is the role of air pollutants, ranging from particulate matter to heavy metals, each bearing its own unique threat to neurological wellbeing. Particulate matter and diverse atmospheric chemicals can penetrate the airways, infiltrate the bloodstream, and traverse the blood-brain barrier, setting the stage for neuroinflammation, neurodegeneration, and cerebrovascular barrier disruptions. Ozone, sulfur oxides, carbon oxides, nitrogen oxides, and heavy metals join the ensemble of neurotoxic agents, each contributing to a complex tapestry of neurological disorders.

While the exact mechanisms linking air pollution and neurological diseases are not yet fully elucidated, evidence strongly suggests their interconnectedness. Neurodegenerative disorders such as AD and Parkinson's disease (PD) have been associated with air pollution, although extensive investigations are warranted.

This Research Topic also speaks about serum zinc levels and lipid profiles in children with spinal muscular atrophy (SMA), a rare genetic neuromuscular disease. Emerging evidence suggests that children with SMA face not only the hallmark motor neuron degeneration but also a lesser-known adversary, dyslipidemia, a harbinger of cardiovascular woes in adulthood. This research topic brings a novel study that probes the relationship between serum zinc levels and lipid profiles in children with SMA, shedding light on an uncharted territory in pediatric metabolic research. The foundation of this study lies in the recognition that SMA is a complex mosaic of muscular and systemic impacts. Distinct metabolic abnormalities, including dyslipidemia, take center stage, prompting investigators to unravel the intricate metabolic tapestry in these young patients.

The study included 112 patients with SMA, from a tertiary children's medical center in China. Dyslipidemia was observed in over 50% of the cohort, emphasizing the critical need for addressing metabolic health in these children. The relationship between serum zinc levels and lipid profiles was investigated with anthropometric, clinical, and biochemical data. Intriguingly, higher serum zinc levels were positively linked with elevated high-density lipoprotein cholesterol (HDL-C) and apolipoprotein A1 (APO A1) levels. This discovery, independent of factors such as age, sex, and body composition, ushers in a fresh perspective on the complex interplay between elemental nutrition and metabolic health. As researchers and clinicians alike reflect on these findings, the vista of future studies and potential interventions broadens (Long et al.).

Fan et al. focus on the neural underpinnings of innate fear. The intricate web of neural circuits underlying fear responses has captivated neuroscientists for decades, and recent advancements have provided new avenues to unravel its complexity. In this editorial, we also discuss a study that investigates the electrophysiological intricacies of the medial amygdala (MA) in processing innate fear. Through the innovative use of microelectrode arrays (MEA) and nanomaterial modifications, this study provides unprecedented insights into the mechanisms orchestrating fear-related neural activity.

Fear, an evolutionarily conserved emotion, drives survival behaviors in both humans and animals. While much is known about the brain's involvement in fear responses, the specific neural pathways and mechanisms remain elusive. The study addresses this by focusing on innate fear, a type of fear that is hardwired and not reliant on learned associations. The predator odor-induced innate fear response in rodents serves as an exemplary model to explore fear-related neural circuits. The augmentation of high throughput MEA detection through the integration of metal nanoparticles and conductive polymers showcases the study's commitment to pushing technological boundaries. The reduction in impedance and phase delay of the electrodes elevate the signal-to-noise ratio, thereby enabling precise neural signal detection even amidst background noise. This study embraces a comprehensive approach by implanting the MEA electrode in the MA of mice to enable synchronous recording of electrophysiological activity and behavioral responses under 2-methyl-2-thiazoline (2MT)-induced fear, thus allowing real-time exploration of the neural dynamics accompanying fear-related behavior. Upon 2MT exposure, strong defensive behavior was observed, concomitant with elevated average spike firing rates and local field potential (LFP) power of MA neurons. The study revealed the presence of two distinct neuron types that displayed a nuanced and complex interplay in regulating innate fear processing, emphasizing the role of MA in innate fear responses. Such studies can indeed shed light on the neural basis of psychiatric disorders such as major depressive disorder, general anxiety disorder, and post-traumatic stress disorder.

These manuscripts not only contribute to advancing knowledge in their respective fields but also effectively demonstrate unique strengths, ranging from uncovering novel insights into AD and innate fear processing to addressing the impact of air pollution on neurological disorders and exploring the relationship between serum zinc levels and lipid profiles in SMA. Each of these studies opens avenues for further exploration and potential therapeutic interventions.

## Author contributions

MP: Writing—original draft. MRB: Writing—review and editing. KV: Writing—review and editing. DT: Writing–review and editing.

